# Limited acclimation of early life stages of the coral *Seriatopora hystrix* from mesophotic depth to shallow reefs

**DOI:** 10.1038/s41598-022-16024-6

**Published:** 2022-07-27

**Authors:** Rian Prasetia, Frederic Sinniger, Takashi Nakamura, Saki Harii

**Affiliations:** 1 Sesoko Station, Tropical Biosphere Research Center, University of the Ryukyus, Sesoko 3422, Motobu, Okinawa 905-0227 Japan; 2 Faculty of Science, University of the Ryukyus, Nishihara, Okinawa 903-0213 Japan

**Keywords:** Ecology, Ecology

## Abstract

Mesophotic coral ecosystems (MCEs, reefs between 30 and 150 m depth) have been hypothesized to contribute to shallow reef recovery through the recruitment of larvae. However, few studies have directly examined this. Here we used mesophotic colonies of *Seriatopora hystrix*, a depth generalist coral, to investigate the effect of light intensity on larval behavior and settlement through ex situ experiments. We also investigated juvenile survival, growth, and physiological acclimation in situ. Bleached larvae and a significant reduction in settlement rates were found when the mesophotic larvae were exposed to light conditions corresponding to shallow depths (5 and 10 m) ex situ. The in situ experiments showed that mesophotic juveniles survived well at 20 and 40 m, with juveniles in shaded areas surviving longer than three months at 3–5 m during a year of mass bleaching in 2016. Juvenile transplants at 20 m showed a sign of physiological acclimation, which was reflected by a significant decline in maximum quantum yield. These results suggest that light is a significant factor for successful recolonization of depth-generalist corals to shallow reefs. Further, recolonization of shallow reefs may only occur in shaded habitats or potentially through multigenerational recruitments with intermediate depths acting as stepping stones.

## Introduction

Severe coral bleaching events caused by high surface seawater temperature have repeatedly occurred over the last few decades, leading to degradation and loss of diversity in coral reefs^1^. However, some reef habitats may act as a shelter from stressors, for instance the deeper parts of the reef, known as mesophotic coral ecosystems (MCEs), since these habitats have lower exposure to ultraviolet radiation and fluctuations in temperature compared to shallower reefs^2,3^. MCEs, characterized by limited light intensity (≤ 10% of surface irradiance), occur at depths below 30–40 m and extend to over 150 m, depending on the region^4^. The deep reef refugia hypothesis (DRRH) asserts that MCEs are protected from disturbances that affect shallow-water reefs and could act as a larval source for shallow reefs^2,5^. However, MCEs may not be entirely protected from natural and human threats^6,7^ and contrasting arguments on the role of MCEs as a larval source for shallow reef recovery have been discussed^8–10^.

Larval dispersal and recruitment of corals are essential for coral reefs to maintain and renew their populations^11,12^. Some studies support the DRRH, suggesting larval dispersal may occur from mesophotic to shallow reefs. For instance, population genetic studies suggest that larval migration between mesophotic and shallow reefs varies among reef sites and species^13,14^. Studies on mesophotic coral reproductive biology^9^ and larval dispersal modeling^8^ further support the potential for mesophotic corals to act as larval sources to recolonize shallow reefs. However, these studies are based on indirect evidence of larval migration, and currently there is no information on the tolerance of larvae and juveniles from mesophotic corals to the environmental conditions of shallow reefs.

After dispersal, coral larvae need to acclimatize to their settlement environments. Since mesophotic larvae dispersing to shallow reefs will be exposed to intense light, photo-acclimation responses are essential for their survival. In this context, the tolerance of mesophotic corals to shallow water conditions has been shown for adult colonies in several studies^15–17^. For instance, altered maximum quantum yield (*Fv/Fm*), algal density, and chlorophyll pigments were observed in the surviving adult corals *Stylophora pistillata* (Esper, 1792)^15^ and *Fimbriaphyllia* (formerly *Euphyllia*) * paradivisa* (Veron, 1990)^16,17^ from mesophotic depth when transplanted to shallow reefs. These adjustments are vital for corals to prevent the loss of photosynthetic activity of the algal symbionts after exposure to an excessive amount of absorbed light energy^18,19^. However, larvae and juveniles may be more flexible to environmental changes. For instance, coral larvae^20^ and juveniles^21^ have the flexibility to associate with multiple algal symbiont types that shape their survival and growth. Hence, their physiological responses to depth transplantation should be tested.

*Seriatopora hystrix* Dana 1846*,* is a widespread scleractinian coral in the Indo-Pacific region. This coral is an ideal species model since it is abundant at an MCE in Okinawa^22,23^ while it disappeared locally in a nearby shallow reef following bleaching events in 1998^24^ and 2001 with no recovery in the shallow reef observed until 2010^25^ and later^26^. Here we examined the effect of light on mesophotic coral larval behavior and settlement in a laboratory experiment as well as on the survival, growth, and physiological acclimation of juveniles in a field experiment. These experiments clarify whether coral larvae from mesophotic depth (from ca. 40 m depth) can settle in shallower reefs and whether the settled juveniles can survive and grow in this environment.

## Results

### Light and seawater temperature at different depths

**Figure 1:**
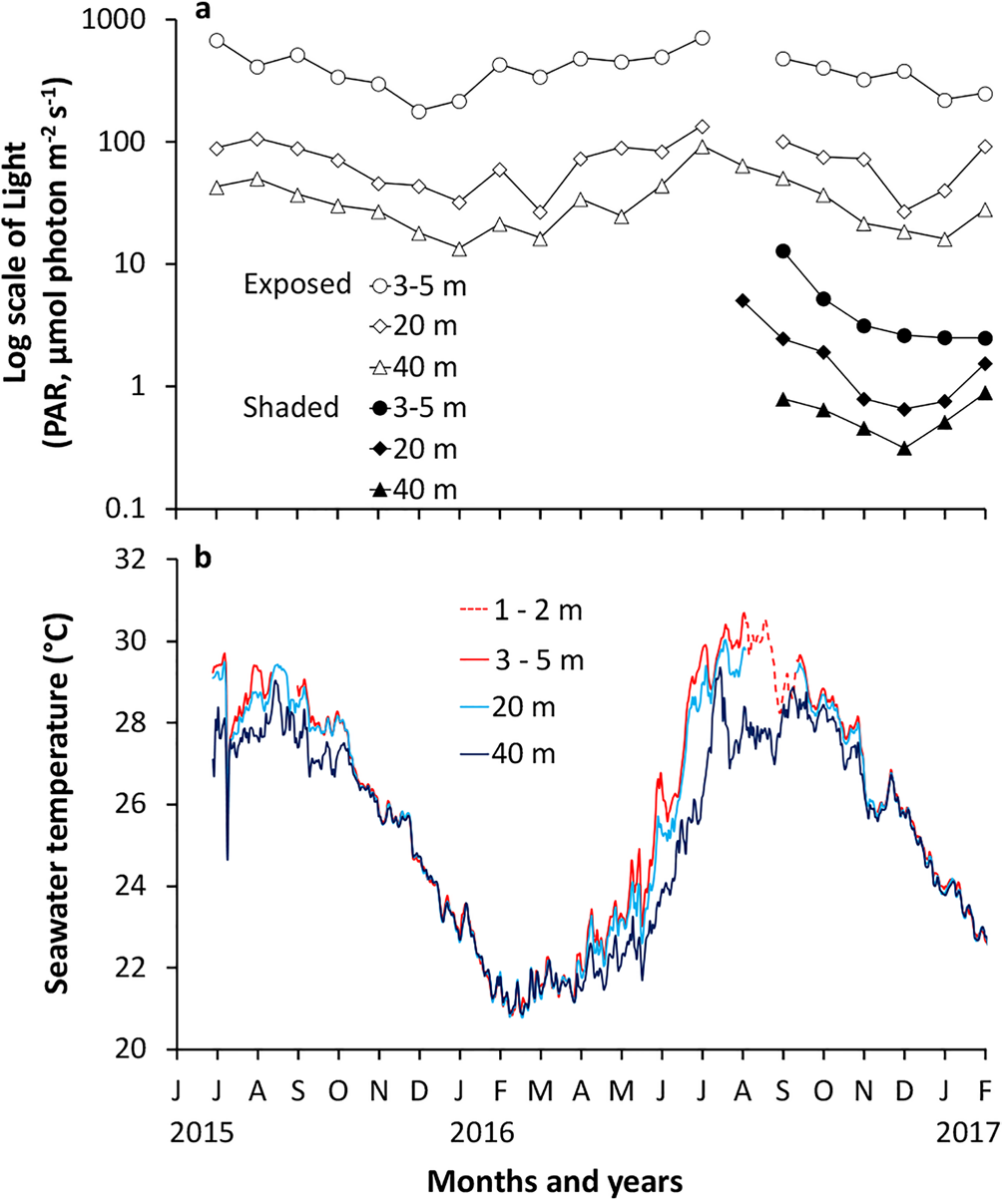
(**a**) Mean of daily maximum irradiance (PAR, Photosynthetically Active Radiation) per month at exposed and shaded orientation, and (**b**) Mean daily seawater temperature (°C) in different reef habitats (1–2, 3–5, 20, and 40 m depths) between June 2015 and February 2017 (mean ± SE). Light loggers at shaded orientation were installed between August 2016 to February 2017. A temperature logger at 1–2 m depth was installed from August to September 2016.

In 2015 and 2016, mean maximum daily light intensities (± SE) in exposed orientation at 3–5, 20, and 40 m depths were 401.8 ± 22.2, 71.4 ± 2.7, and 34.5 ± 1.3 µmol photon m^−2^ s^−1^, respectively (Table 1; Fig. 1a). In shaded environments, the light intensities (± SE) were 4.8 ± 0.3, 1.9 ± 0.1, and 0.6 ± 0.04 µmol photon m^−2^ s^−1^ at the depths of 3–5, 20, and 40 m, respectively (Table 1; Fig. 1a).

**Table 1:** Mean daily maximum irradiance (PAR, Photosynthetically Active Radiation) between June 2015 and February 2017 and mean seawater temperature during the crucial acclimation period (i.e., the first month) of coral juvenile transplant experiments in August–September 2015 and 2016 at the depths of 3–5, 20, and 40 m in exposed and shaded orientations.

Depth	Orientation	Mean maximum daily irradiance (µmol quanta m^−2^ s^−1^)	Mean seawater temperature (°C) in August—September 2015	Mean seawater temperature (°C) in August—September 2016
3–5 m	Exposed	401.8	28.4	29.4
	Shaded	4.8		
20 m	Exposed	71.4	28.5	28.9
	Shaded	1.9		
40 m	Exposed	34.5	27.7	28.0
	Shaded	0.6

During the first month of juvenile transplant experiments (from August to September), the mean seawater temperatures (± SE) were 28.4 ± 0.02, 28.5 ± 0.02, and 27.7 ± 0.02 °C at 3, 20, and 40 m, respectively in 2015 and they were 29.4 ± 0.02, 28.9 ± 0.03, and 28.0 ± 0.02 °C at 1–5, 20, and 40 m, respectively in 2016 (Table 1; Fig. 1b). In 2016, high (> 3 °C) temperature differences (hourly average) between shallow and mesophotic depths were observed day and night. The temperature differences lasted for most of planula release periods in July (between 18 and 28 July 2016) and August (between 15 and 20 August 2016)^9^. Seawater temperature was relatively similar at all depths from November to February in both years of juvenile transplant experiments.

### Larval behavior, settlement, and survival (laboratory experiment)

Regardless of the light conditions, planulae were mostly found at the bottom layer of the 80-cm tall acrylic columns, averaging between 68% (40 m) and 79% (5 m) (Supplementary Fig. 1). The descending speeds (downward swimming) of the planulae, between 1.6 mm s^−1^ (20 m) and 2.1 mm s^−1^ (40 m), were faster than the ascending speeds (upward swimming), between 0.5 mm s^−1^ (40 m) and 0.7 mm s^−1^ (20 m). There was no significant effect of light conditions on larval swimming speed at each direction (One-Way ANOVA, downward, *F* = 0.495, *df* = 3, P = 0.688; upward, *F* = 0.445, *df* = 3, P = 0.728).

**Figure 2:**
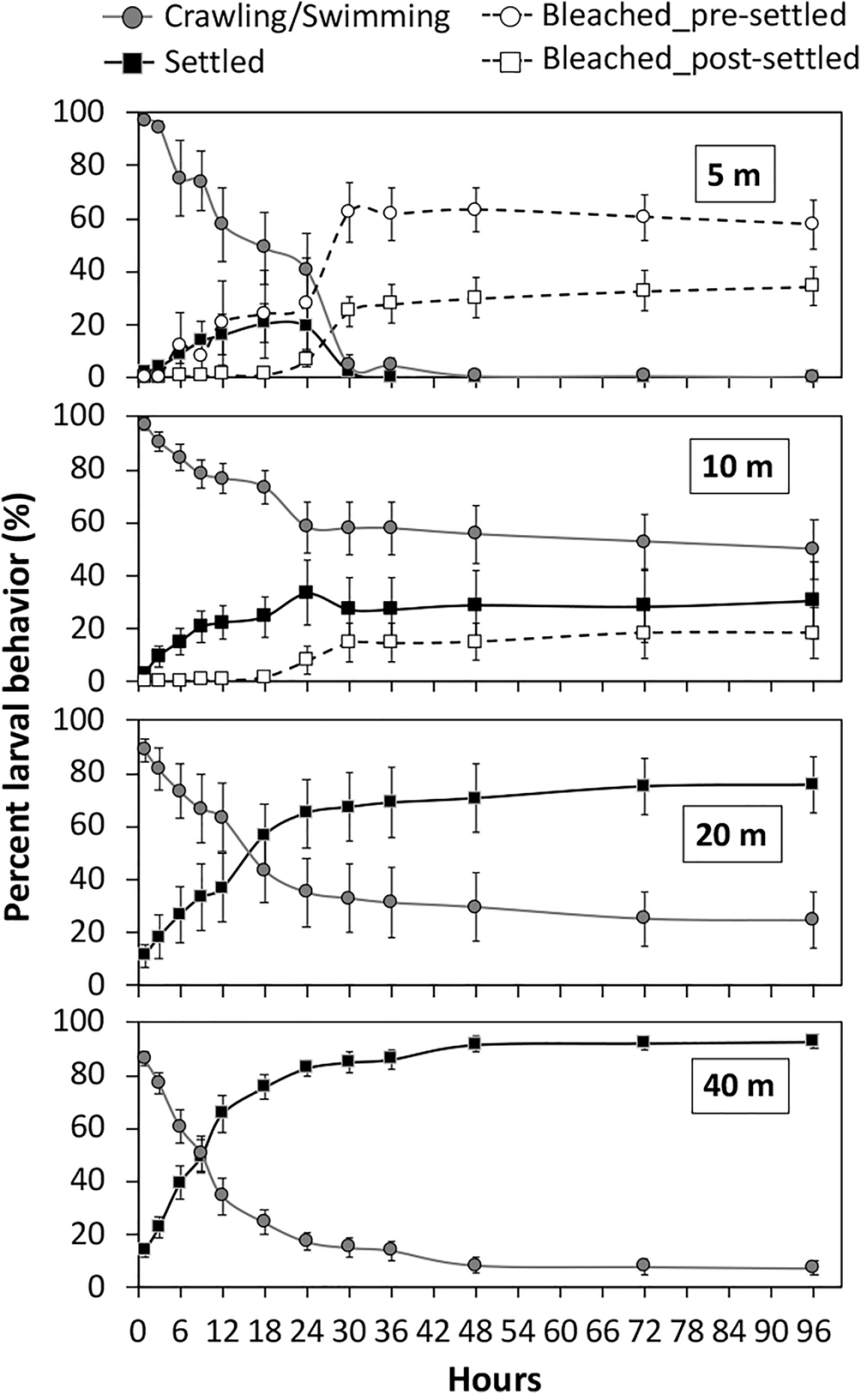
Mean number of larval behavior (± SE; n = 6) of *S. hystrix* larvae from mesophotic colonies. Larvae identified as settled, crawling/swimming, bleached (pre- and post-settled), and survived (pre- and post-settled) under light conditions representing the depth of 5, 10, 20 and 40 m in 2016.

The planulae were actively crawling and settled rapidly within hours after release under control (i.e., 40 m depth) light conditions (Fig. 2). Percent of settled larvae differed significantly between light conditions both in 2015 (Mann–Whitney U, P = 0.004, pairwise comparison: 40 m > 10 m) and 2016 (One-Way ANOVA, *F* = 9.008, *df* = 3, P = 0.001; Fig. 2). The percentage of settled larvae reduced significantly in 5 and 10 m light conditions compared to control, while the percentage of settled larvae in 20 m light condition was similar (40 m = 20 m ≥ 10 m = 5 m lights; *Bonferroni*, P = 0.001). All larvae survived in 40 and 20 m conditions, while in 10 and 5 m conditions, survival rates of both crawling/swimming and settled larvae reduced by 1.1 and 7.8%, respectively (Fig. 2). In addition, 16.9 and 100% of the surviving pre- and post-settled larvae became pale or bleached when exposed to 10 and 5 m light conditions, respectively (Supplementary Fig. 2).

### Survival and growth rate of juvenile (field experiment)

**Figure 3:**
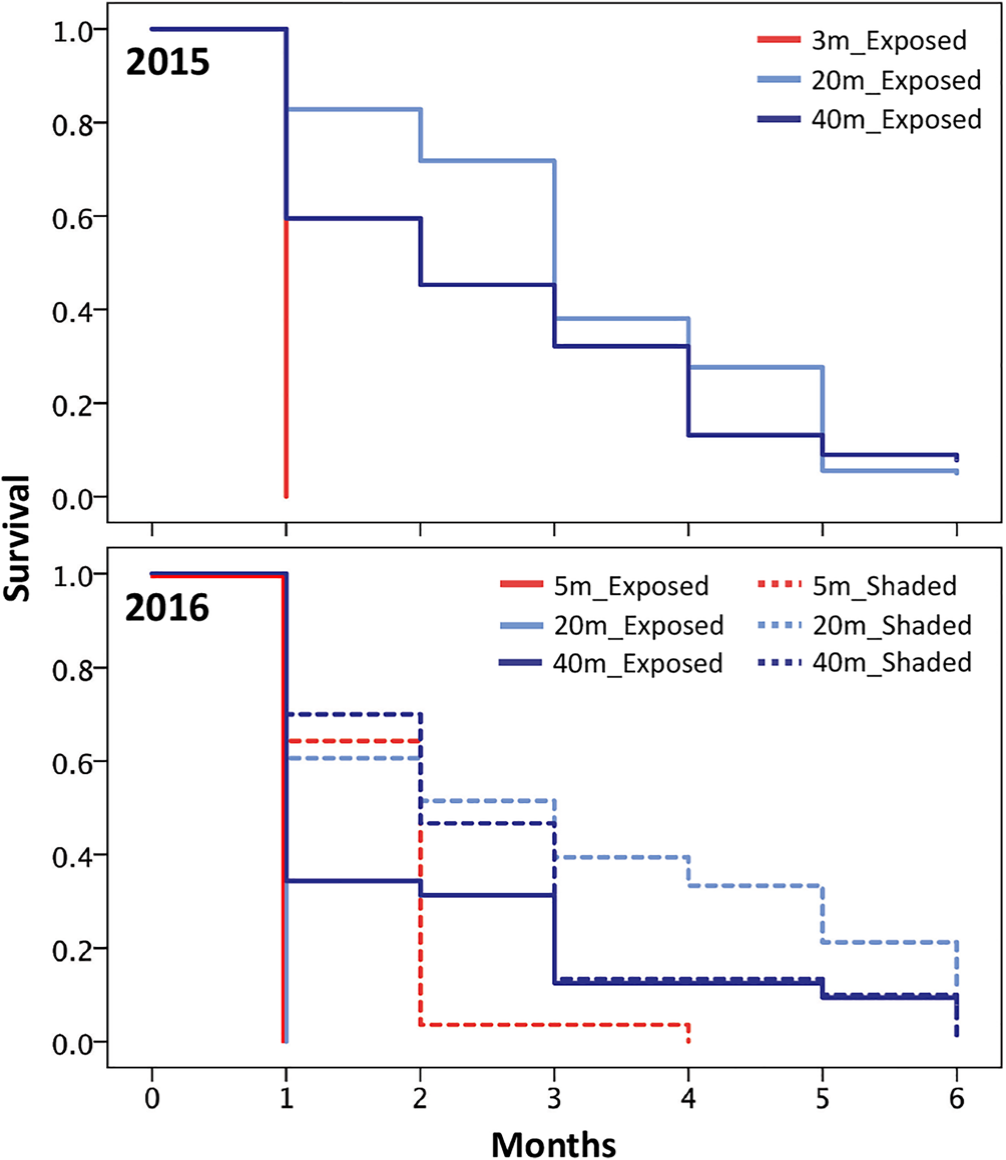
Survival of *S. hystrix* juveniles over six months in 2015 experiment from August 2015 to February 2016 (3, 20, and 40 m depth; only in exposed orientation) and in 2016 experiment from August 2016 to February 2017 (5, 20, and 40 m depth; exposed and shaded orientation).

Most juveniles in exposed orientation at 3–5 m (in 2015, 2016) and 20 m (in 2016) did not survive the first month (Fig. 3). In 2015, the survival of juveniles at 3–5 m depth (0%) was significantly lower than other depths (i.e., 20 and 40 m) (Mantel-cox log-rank test*,* P < 0.001; Fig. 3; Supplementary Table 1). Depth also influenced juvenile survival at the deeper sites in an exposed orientation in 2015, where the 20 m juveniles had significantly lower survival (9.5%) than the 40 m control juveniles (11.1%) after six months (Mantel-cox log-rank test*,* P = 0.005; Fig. 3; Supplementary Table 1). In 2016, juveniles at 20 m in a shaded orientation (12.1%) and the control juveniles (40 m depth, exposed orientation; 6.3%) survived after six months, while the juveniles at 3–5 m shaded orientation only survived three months (3.6%) (Fig. 3). Importantly, orientation influenced juvenile survival, where the survival at 3–5 m and 20 m in a shaded orientation was similar to survival at 40 m in an exposed orientation (Mantel-cox log-rank test; P > 0.05; Supplementary Table 1).

**Figure 4:**
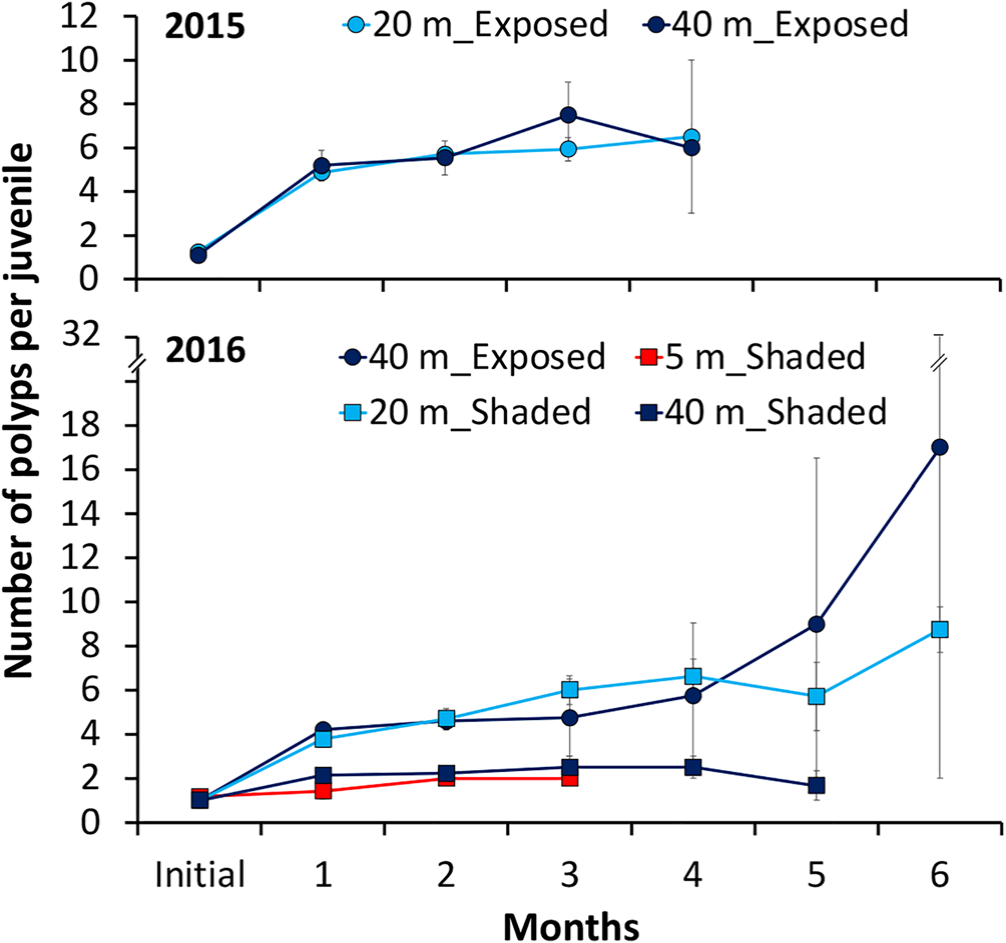
Mean number of *S. hystrix* polyps per juvenile (± SE) in 2015 experiment from August to December 2015 at 20 and 40 m depth and in 2016 experiment from August 2016 to February 2017 at 5, 20, and 40 m depth in exposed orientation (circles) and shaded orientation (squares).

In 2015, the number of polyps in juveniles in an exposed orientation at 20 and 40 m increased (Fig. 4), and the mean geometric diameter also increased (Supplementary Fig. 3), although differences were not significant. An average (± SE) of 5.7 ± 0.33 and 5.5 ± 0.78 polyps per juvenile was observed after two months at 20 m and 40 m, respectively (Student’s t-test, t = -0.209, df = 53, P = 0.835). Mean geometric diameter (± SE) of juveniles at 20 and 40 m frames was 1.52 ± 0.04 and 1.44 ± 0.06 mm, respectively, with no significant difference observed (Student’s t-test, t = -0.911, df = 53, P = 0.366, Supplementary Fig. 3).

**Figure 5:**
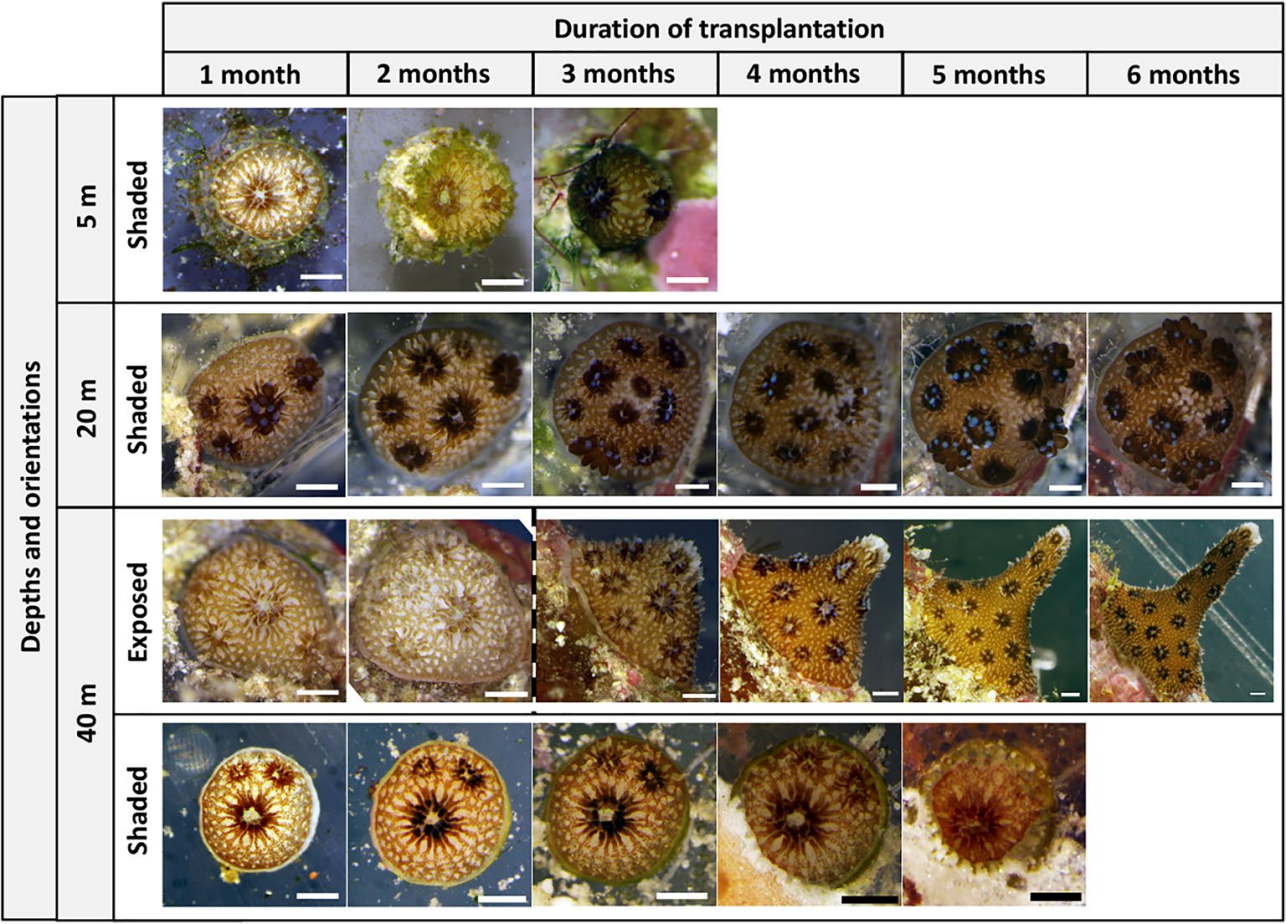
*S. hystrix* juveniles from mesophotic colonies transplanted at 5 m shaded orientation, 20 m shaded orientation, and 40 m exposed and shaded orientation over six months transplantation from August 2016 to February 2017. For the 40 m exposed orientation, the juvenile at the 1st and 2nd month is different individual with the juvenile from the 3rd to 6th month of transplantation. Scale bars = 400 µm.

Similarly, in 2016, the number of polyps in juveniles increased in both exposed and shaded orientations at all depths (Figs. 4, 5). After two months, the shaded juveniles at 20 m (mean ± SE of 4.7 ± 0.46 polyps per juvenile) and the exposed juveniles at 40 m (mean ± SE of 4.6 ± 0.40 polyps per juvenile) had a significantly greater number of polyps per juvenile than the juveniles at 40 m in a shaded orientation (mean ± SE of 2.2 ± 0.20 polyps per juvenile) (Kruskal–Wallis followed by Dunn tests, Chi-squared = 17.92, df = 2, P < 0.001). The shaded juveniles at 20 m had a significantly larger mean geometric diameter (1.40 mm) compared to the 40 m juveniles in exposed (1.24 mm) and shaded orientations (1.23 mm) (Kruskal–Wallis followed by Dunn tests, Chi-squared = 7.43, df = 2, P = 0.020).

### Maximum quantum yield, algal density, and chlorophyll pigments of juveniles (field experiment)

**Figure 6:**
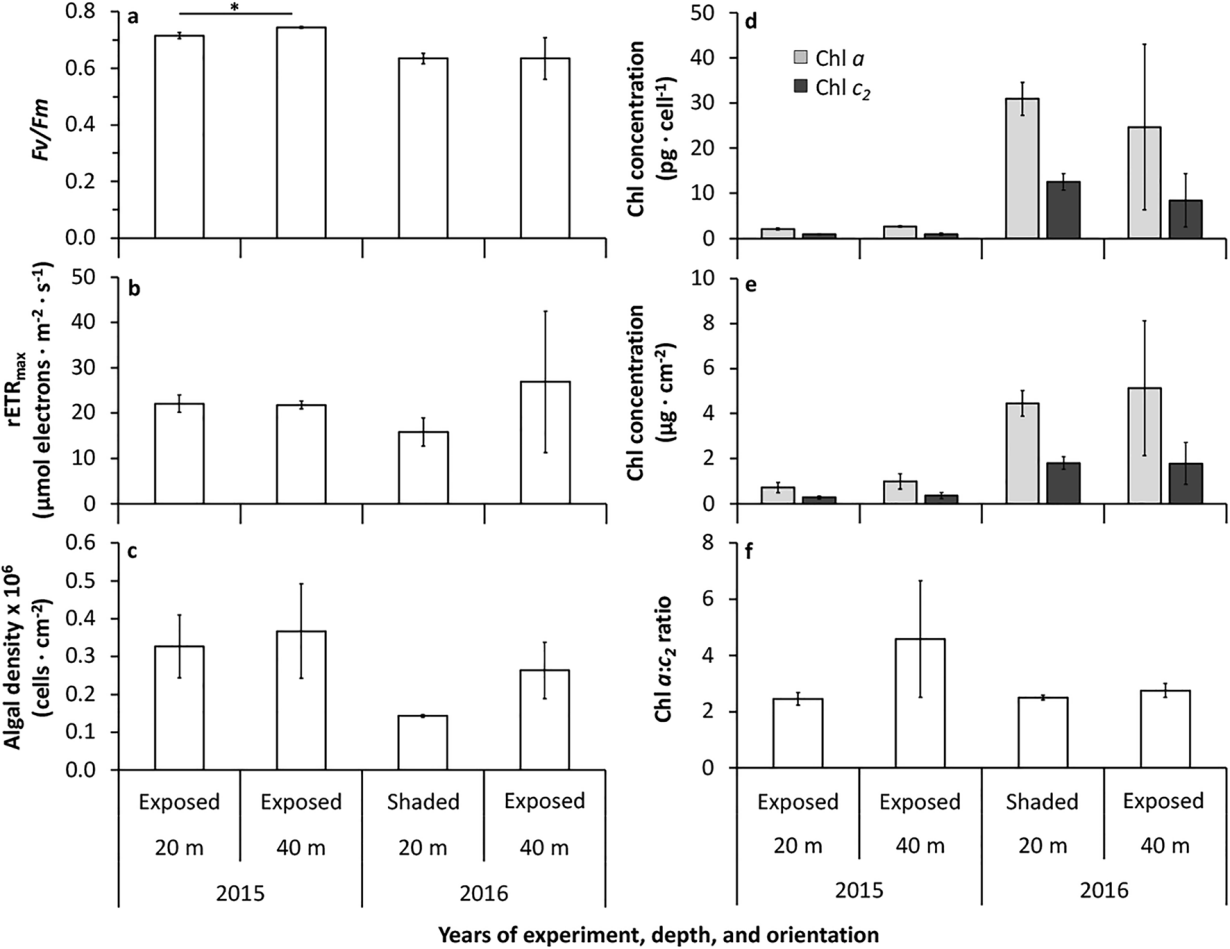
Maximum quantum yield of Photosystem II (*Fv/Fm*) (**a**), rETRmax (**b**), algal density per surface area (**c**), chlorophyll (*a* and *c*_2_) per algal cell (**d**) and per surface area (**e**), and the ratio of chlorophyll *a* and *c*_2_ (**f**) of the surviving *S. hystrix* juveniles at the end of the experiment (after 6 months) in 2015 (20 and 40 m depth both in exposed orientation) and in 2016 (20 m shaded orientation and 40 m exposed orientation) (mean ± SE). The asterisk indicates a significant difference (*p* = 0.028).

Among the surviving juveniles at the end of 2015 experiment, those at 20 m had significantly lower *Fv/Fm* (0.715 ± 0.01; mean ± SE; n = 5) compared to juveniles at 40 m (0.744 ± 0.003; n = 5), while similar maximum relative electron transport rates (rETRmax) were observed (Welch’s t-test, P = 0.049 for *Fv/Fm*; P = 0.898 for rETRmax; Fig. 6a, b; Supplementary Table 2). In 2016, shaded juveniles at 20 m had a *Fv/Fm* of 0.635 ± 0.02 (mean ± SE; n = 4) and rETRmax of 15.8 ± 3.1 µmol electrons m^−2^ s^−1^ (mean ± SE; n = 4), while exposed juveniles at 40 m had a *Fv/Fm* of 0.635 ± 0.07 (n = 2) and rETRmax of 26.9 ± 15.6 (n = 2), respectively (Fig. 6a, b). No statistical comparison was conducted in the 2016 experiment, due to insufficient number of surviving replicates.

Algal density, chlorophyll *a* and *c*_2_ per cell and surface area, and chlorophyll ratio (*a*:*c*_2_) were similar for juveniles at 20 and 40 m in 2015 (Fig. 6c–f, Supplementary Table 2). In 2016, symbiont density in the shaded juveniles at 20 m and exposed juveniles at 40 m was 1.4 and 2.6 × 10^5^ cells cm^−2^, respectively. Concentrations of chlorophyll *a* and *c*_2_ per cell were 31.0 and 12.5 pg cell^−1^, respectively, for those shaded at 20 m, while those exposed at 40 m had 24.7 and 8.4 pg cell^−1^, respectively (Fig. 6d). An insufficient number of surviving replicates in 2016 prevented statistical comparisons.

## Discussion

Our results provide several insights into the potential recovery of *S. hystrix* in shallow reefs by means of larvae released from colonies living at mesophotic depths. In our laboratory-based experiments, we observed most larvae rapidly crawling at the bottom of the acrylic columns used, settling within 24 h; suggesting most *S. hystrix* larvae from mesophotic colonies settle close to their parent colonies at mesophotic depths. This behavior is consistent with previous observations on this species^9,27^. However, our results also suggest some larvae could disperse far from their natal reefs since up to 16% of the larvae remained in the water column or at the surface (Supplementary Fig. 1), and up to 9.7% of the larvae kept swimming four days after release (Fig. 2). This suggests that a non-negligible portion of *S. hystrix* larvae from mesophotic depth may swim vertically or are passively transported by currents in the water column to find a suitable place to settle further away.

The upward swimming behavior of mesophotic coral larvae and their position in the water column could partly contribute to vertical larval dispersal to shallow reefs. In the present study, since the upward swimming speed of *S. hystrix* larvae from mesophotic depth was 0.6 mm s^−1^, in theory, larvae could reach the surface from 40 m depth after 18 h. This lies within the timeframe of larval settlement competency periods. However, the swimming speed of coral larvae is much slower than horizontal currents in the reefs^28^. Larval dispersal distance is primarily determined by the larval competency period, their position in the water column and the coincident water currents^29–31^. The longer larvae stay in the water column, the more likely they will disperse. In the case of mesophotic larvae, water movement will also affect their vertical migration. For example, off North Carolina’s coast, upwelling influenced the shoreward migration of larval invertebrates and fish^32^. No information is available on upwelling around Okinawa during the coral reproductive season. However, at the time of larval release, typhoons could create enough vertical water movement to potentially transport *S. hystrix* larvae from mesophotic depths to shallow reefs, although the frequency and paths of strong storms vary across years^9,33^. This stochastic connectivity may explain the absence of genetic partitioning within the water column for *S. hystrix* in Okinawa^26^. Future studies on current patterns during spawning/larval release are necessary to understand larval dispersal processes from mesophotic reefs.

Mesophotic larvae that are dispersed to shallower areas of the reef face several challenges to their survival. The laboratory experiments showed that settlement rates of *S. hystrix* larvae from mesophotic colonies were reduced when exposed to light conditions corresponding to depths shallower than 20 m (i.e., 10 and 5 m) (Fig. 2). This supports previous findings where the larvae of shallow corals preferred to settle in similar light conditions as parental colonies^34,35^. In addition, most of the pre- and post-settled larvae partially or completely bleached under light conditions representing depths shallower than 20 m (Supplementary Fig. 2). The vertical transmission of algal symbionts into larvae from mesophotic corals like *S. hystrix* might be unfavorable for larvae dispersed to shallower water since the higher light conditions increased DNA damage in the symbiont algae^36^. Likewise, excess algal symbionts increases the susceptibility of adult corals to bleaching^37^. Therefore, in shallow reefs, settlement of larvae from mesophotic corals may be limited to shaded microhabitats such as steep spurs or overhangs*.* However, it is essential to note that light conditions cannot be solely interpreted as light intensity alone due to differences in the light spectrum used in our experiments; hence, further investigation into the independent effect of light quantity and quality is required.

In addition to high light stress, *S. hystrix* larvae from mesophotic depth will likely be exposed to thermal stress when dispersing to shallow reefs. In 2016, many corals bleached between 0 to 20 m depth in Okinawa^38,39^ (pers. Obs.), where high (> 3 °C) temperature differences between shallow and mesophotic depths occurred for the majority of the planula release period. Elevated temperatures (> 3 °C above average seasonal temperature) can lower larval survival and reduce dispersal^40^. In the case of *S. hystrix* from mesophotic depths in Okinawa, high temperature discrepancy between shallow and mesophotic depths during thermal stress events will likely reduce settlement success in shallow reefs.

While laboratory experiments on larvae focused on light stress, field experiments on juveniles combined light and thermal stresses. The overall survival of juveniles significantly increased with depth, and those in a shaded orientation showed higher survival than juveniles in exposed orientations (Supplementary Table 1). As observed for the larvae in laboratory experiments, the high light intensity may cause stress on juveniles at 3–5 and 20 m depths in exposed orientation (light intensity was ~ 12 and ~ 2 fold higher than 40 m depth, respectively). Similarly, for adult corals, most of the low light-adapted corals transplanted to high light environments showed bleaching^41^ and high mortality^42,43^ (but see cases for the *S. pistillata*^15^ and *F. paradivisa*^16^ for contrasting responses).

The survival of juveniles from mesophotic depth in shallower reefs was also affected by substrate orientation. In the 2016 field experiments, in shallower reefs (i.e., 3–5 and 20 m depths), most juveniles survived when in a shaded orientation (Fig. 3), i.e. in low light environments created by crevices and overhangs. These observations support previous studies showing the frequent occurrence of juveniles on shaded or vertical surfaces in shallow reefs^44,45^. Such surfaces also provide protection from sediment accumulation^46^ and fish grazing^47^. Conversely, almost all juveniles at 3–5 m and those at 20 m in an exposed orientation died within the first month of transplantation (Fig. 3). This mortality corresponds with exceptional thermal stress. Indeed, around Sesoko Island, sea surface temperature in 2016 was the second-highest in the last three decades after the 1998 bleaching event^39^. Since mortality in adult corals has been attributed to thermal and high light stresses^48^, this combination most likely affected the survival of *S. hystrix* from mesophotic depth in shallow and intermediate depths in 2016.

Surprisingly, the coral juveniles that survived at 20 m depth showed significantly increased growth rate at 20 m, or remained relatively similar, to those at 40 m, both at the exposed and shaded orientation (Fig. 4; Supplementary Fig. 3). A similar result has been reported previously, where juveniles of *Stylophora kuehlmanni* Sheer and Pillai 1983 were almost twice as large at 5 m as those at their natural depth of 45 m^49^. Likewise, adult colonies of *S. hystrix* (Pers. Obs.), *S. pistillata*^15^, and *Orbicella franksi* (Gregory 1895)^50^ grew significantly faster at shallow reefs (< 20 m) than those at MCEs. In these cases, higher light intensity in the shallower reefs may have enhanced calcification in corals by light activation^51^. However, this is unlikely to have occurred in our study since a significant increase in growth rate was only found at the 20 m shaded orientation with extremely low light intensity (~ 18 fold lower than 40 m exposed orientation). A possible explanation is that warmer seawater temperature (~ 0.5 °C warmer at 20 m than at 40 m) during the first two months of juvenile transplant experiments enhanced juvenile growth^52,53^. The juvenile growth rates observed in our study indicate that the intermediate depth of 20 m is adequate for the successful recruitment of *S. hystrix* from mesophotic depth*.*

Photoacclimation responses are also likely to play an essential role in juvenile survival in shallow reef environments. Here, the response of the algal symbiont to the higher light conditions can be seen through the decrease in maximum quantum yield, *Fv/Fm* (Fig. 6a). It should be noted that measurements of *Fv/Fm* were performed in the laboratory and, as such, changes in seawater temperature during the transfer from the field sites could have affected the results. To minimize this, samples were carefully transferred under dark conditions and immediately measured upon arrival to the laboratory. Minor variations were observed between samples (Fig. 6a), likely due to slight differences in light intensity between 20 and 40 m depth at the time of measurement (the light intensity differences between 20 and 40 m was ~ 37 µmol photon m^−2^ s^−1^), however the seawater temperature was similar at both depths. The differences in light intensity between 20 and 40 m depths may also be responsible for similar variations in the other indicators measured (algal density and chlorophyll concentrations). Other studies have also reported a decrease in *Fv/Fm* as one of the photoacclimatory processes of MCEs adult colonies of *S. pistillata* (from ~ 0.67 at 30 m to ~ 0.64 at 3 m) and *F. paradivisa* (from 0.66 at 50 m to 0.49 at 5 m) when transplanted to shallower depths^15,17^. A decrease in maximum quantum yield under high light conditions reflects potential damage to photosystem II or adaptation to reduce photo-oxidative damage to the photosynthetic apparatus in the symbiotic algae within corals^54^. Thus, adjustment of maximum quantum yield, *Fv/Fm,* in the symbiont can be considered a strategy for coral juveniles from mesophotic depth to acclimate to different light environments.

Green fluorescent proteins (GFPs) in corals have been suggested to play a role in photoprotective mechanism^55^, avoidance from predators^56^, and prey attraction^57^. While no study on corals from mesophotic depths has investigated fluorescent proteins in juveniles, a few studies focused on adults^58–61^. However, no specific conclusion could be drawn regarding the potential role of fluorescent proteins in mesophotic corals even though ~ 70% of corals fluoresced over the entire part of the corals observed^60^. In the Red Sea, mesophotic *Galaxea fascicularis* (Linnaeus 1767) from mesophotic depth increased its GFPs when exposed to light representing 3 and 20 m depth, suggesting a photoprotective function of GFPs^59^. In the present study, the juveniles at both 40 and 20 m (exposed and shaded orientation) had GFP with similar fluorescent characteristics (see ESM). Therefore, GFP for juveniles in a low light environment is likely to have other roles than photoprotection. Further studies are needed to quantify the intensity of coral fluorescence at mesophotic depth, investigating the role of fluorescent proteins as the coral’s defense mechanism at high light intensity during acclimation to shallow reefs.

In terms of recolonization of shallow reefs, our results showed that a small portion (up to 16%) of *S. hystrix* larvae from mesophotic colonies might disperse to shallower reef habitats. A shorter reproductive season and smaller planula size compared to their shallow counterparts^9^ also supports limited dispersal. Moreover, as light conditions at shallower depths impede direct recolonization of coral larvae from mesophotic depth, rugosity or tridimensional complexity of the shallow reef will play an essential role for mesophotic larvae by providing suitable shaded habitats. Around our study site at intermediate depth (~ 20 m), in 2015 and 2016 a few small colonies of *S. hystrix* were observed, and more recently, the occurrence of *Seriatopora* colonies at 20 m and 10 m appears to be increasing (pers. obs.). In 2021, a single, well-developed colony at 4.8 m was observed within the field experiment area (pers. obs.). No evidence yet whether those new colonies at a shallower depth originated from settlement of larvae from mesophotic colonies. Our results, and the absence of genetic structure related to depth between shallow and mesophotic *Seriatopora* in the region^26^, suggest that 20 m depth may act as a stepping stone to connect mesophotic corals to shallow reefs. We suggest that recolonization from mesophotic depths to shallow reefs occurs through multigenerational recruitment over a long-term period. However, anthropogenic stressors exacerbating coral reefs’ degradation^62^ will limit the potential of mesophotic corals to reseed shallow reefs. Indeed, *S. hystrix* juveniles from mesophotic depth transplanted to shallower depths during the thermal stress event of 2016 suffered high mortality, even at 20 m. If such events occur more frequently in the future^63^, they may reduce the stepping stone capacity of 20 m reef habitats. Given the ecological importance of mesophotic corals, the origin of shallow water colonies and the process for recolonization of these habitats deserves further investigation. To fully understand the contribution of MCEs to shallow reef recovery, future studies should include a range of corals with differing spawning mechanisms and agal transmission modes.

## Methods

### Study sites, coral collection, and environmental measurements

Six to ten mature colonies (> 15 cm diameter) of *S. hystrix* were collected several times between June and August 2015 and 2016 from an MCE site (40 m depth) north of Sesoko Island, Okinawa, Japan (Supplementary Fig. 4, Supplementary Table 3). Each colony was maintained in indoor seawater systems at the Sesoko Station of the University of the Ryukyus (described in^9^). *Seriatopora hystrix* is a brooder whose larvae contain symbiotic algae when released (vertical transmission)^27^ and, in Okinawan mesophotic reefs, *S. hystrix* release larvae monthly from May to August^9^. The larvae released during the peak planula release period^9^ were used for larval behavior and settlement experiments ex situ and juvenile acclimation experiments in situ. They were kept in filtered seawater (0.2 µm) until the experiments to avoid the presence of larvae and symbiotic algae from other species coming through the seawater supply and keep better water quality. In situ juvenile acclimation experiments were conducted at three sites: at the MCE site (40 m; where the original adult parent colonies originated) and at two shallower sites (3–5 m and 20 m, within 750 m from the MCE site; Supplementary Fig. 4). Seawater temperatures and light intensity were measured hourly at each site using loggers (HOBO Pendant/Light Data Logger, Onset Computer Corporation, USA).

### Effect of different light conditions on larval behavior and settlement (laboratory experiment)

Fifty to a hundred larvae colony^−1^ day^−1^ were released on 18–20 July 2016 (5 colonies). They were all pooled in a 10 L tank filled with filtered seawater daily. Larvae were used in the following two experiments within 6 h of release. To examine the larval position in the water column, ten larvae were exposed to light conditions representing environments at 40 m, 20 m, 10 m, and 5 m depths (i.e., 50–60, 250, 450 and 600 µmol quanta m^−2^ s^−1^, respectively) in 80-cm-tall acrylic columns using LED lights (Hydra 52, C2 Development Inc., USA) (Supplementary Table 4). After 10 min, the planulae were counted at the surface (0–2 cm deep), the upper layer (2–40 cm deep), the lower layer (40–78 cm deep), and the bottom layer (0–2 cm above the bottom). The experiment was replicated eight (for 5 and 40 m conditions) or ten times (for 10 and 20 m conditions) using new individuals. In addition, the swimming speeds of larvae were measured by tracking their movement for up to 2 min in each light condition (n = 3, 7, 7, and 8 larvae in total in 5, 10, 20, and 40 m light conditions, respectively). The tracks were traced to transparent plastic sheets on the side of the acrylic column using a marker.

To examine larval settlement and survival, 30 newly released planulae were transferred to 700 ml plastic containers. Plastic settlement plates (circular shape, diameter: 6 cm, circle polyethylene V-type container V-1, AS ONE, Japan) were placed in each container since *S. hystrix* larvae prefer to settle on plastic^9^. The containers were exposed to 13 h:11 h (light:dark) photoperiods under the light environment at 40 m, 20 m, 10 m, and 5 m depths, see above). Six replicates were performed in each light condition. The larvae were counted over four days and classified as (i) settled, (ii) crawling/swimming, or (iii) dead. Pre- and post-settled larval conditions were monitored as healthy or bleached (Supplementary Fig. 2). Seawater temperature was kept at 27 ± 1 °C (i.e., the temperature at 40 m) and was replaced twice a day (i.e., 8:00–10:00 and 18:00–20:00) with 50–60% FSW.

### Juvenile acclimation to different depths (field experiment)

Hundreds of larvae per colony released on 27 July–6 August 2015 (8 colonies) and 18–28 July 2016 (9 colonies) were pooled and kept in a 10 L filtered seawater tank. The plastic settlement plates (same as above) were then placed in the 10 L tank to facilitate settlement. A total of 477 and 181 planulae settled on 29 and 46 settlement plates in 2015 and 2016, respectively (ranging between 1 and 50 planulae per plate). The location of juveniles on the plates was mapped under a dissecting microscope. In both years, plates with 1-week-old juveniles were fixed horizontally to the top of rectangular PVC frames and deployed in August at three depths (3–5, 20, and 40 m sites). In 2015, n = 146, 163, and 168 juveniles on the plates were distributed at 3–5, 20, and 40 m sites, respectively, and all plates were placed upward on the frames exposed to direct sunlight (hereafter, this position is referred to as exposed orientation). In 2016, the plates were placed upward and downward (hereafter referred to as shaded orientation) on the frames (Supplementary Fig. 5). The plates hosted between 28 and 32 juveniles at each depth and orientation.

### Survival and growth of juveniles

Monthly observations were performed to examine juvenile survival and growth in 2015 and 2016. The juveniles were carefully transported to Sesoko Station (in the dark, covered by shade cloth) and maintained under their respective depths’ light conditions. Juvenile survival was examined as either alive or dead based on their color and the presence of coral tissue under a dissecting microscope. The number of polyps and geometric diameter were measured under a dissecting microscope to examine juvenile growth. In 2015, 291 out of 477 juveniles were selected for growth measurements. In 2016, all the juveniles were measured. After the observations (within two days), the juveniles were returned to the originally transplanted depths.

### Maximum quantum yield, algal density, chlorophyll pigments of juveniles

At the end of the experiment in both years (February 2015 and 2016), the juveniles were transferred under dark conditions to the laboratory to measure the maximum quantum yield of the symbiotic algae using a pulse-amplitude modulated chlorophyll fluorometer (Diving-PAM, Walz, Germany). A total of ten juveniles (5 each for 20 m and 40 m exposed orientation; 2015) and six juveniles (4 for 20 m shaded orientation; 2 for 40 m exposed orientation; 2016) were dark-adapted for 30 min before maximum quantum yield (*Fv/Fm*) and maximum relative electron transport rate (rETRmax) measurements (Supplementary Materials and Methods). Soft tissues of each juvenile were then removed using an airbrush. The tissues were then extracted for algal density, chlorophyll pigments (described in^58^) and fluorescent protein analyses (Supplementary Materials and Methods).

### Statistical analyses

Before analyses, the statistical assumptions were verified with Shapiro–Wilk (normality) and Levene’s (homogeneity) tests and transformed if necessary (log, inverse sine, and square root transformation). All statistical analyses were conducted using SPSS (version 11.5).

For laboratory experiments, one-way ANOVA tests were performed to examine differences in larval swimming speeds across light conditions (40, 20, 10, and 5 m) for each downward (n = 9, 9, 12, and 4 measurements, respectively) and upward direction (n = 3, 3, 3, and 2 measurements, respectively). A one-way ANOVA, followed by Bonferroni post hoc tests, was performed to test for significant differences in larval settlement rate after 96 h under different light conditions (40, 20, 10, and 5 m light conditions); the container was the experimental unit (n = 6), the light condition was considered as a fixed factor.

For the field experiments, survival rates of juveniles were estimated using Kaplan–Meier (K–M) survival analysis. Each juvenile was assumed to be independent of the other. Mantel-cox log-rank tests were compared the estimated K-M survival curves among depths in 2015 and depths and orientations in 2016. Pairwise comparisons of Mantel-cox log-rank tests were used to quantify differences in survival curves for a given year. Student’s t-tests were used to detect the significance of the number of polyps and geometric diameter between depths (20 m, n = 44; 40 m, n = 11) at two months in 2015. In 2016, a Kruskal–Wallis test, followed by Dunn post hoc tests, was used to detect significant differences between depths and orientation (20 m shaded orientation, n = 17; 40 m exposed, n = 10; and 40 m shaded, n = 14). Statistical analyses for growth in both years were only performed during the first two months of transplantation due to an insufficient number of surviving juveniles beyond these periods. Welch’s t-tests were used to examine significance between depths (20 and 40 m; both n = 5) in 2015 on maximum quantum yield (*Fv/Fm* and rETRmax) and chlorophyll (*a* and *c*_2_) per surface area and per cell. A Mann–Whitney U test examined the significance of chlorophyll (*a*:*c*_2_) ratio between depths (20 and 40 m; both n = 5).

### Ethics approval

Coral colonies were sampled under permits issued by Okinawa prefectural government, Japan (No. 27-28 and 28-21).

## Supplementary Information

Supplementary Information.

## Data Availability

All data needed to evaluate the conclusions are present in the paper and/or the Supplementary Materials. The raw data analyzed in this study are available from the corresponding author on request.
