# Author Correction: Limited acclimation of early life stages of the coral *Seriatopora hystrix* from mesophotic depth to shallow reefs

**DOI:** 10.1038/s41598-022-20029-6

**Published:** 2022-09-12

**Authors:** Rian Prasetia, Frederic Sinniger, Takashi Nakamura, Saki Harii

**Affiliations:** 1 Sesoko Station, Tropical Biosphere Research Center, University of the Ryukyus, Sesoko 3422, Motobu, Okinawa 905‑0227 Japan; 2 Faculty of Science, University of the Ryukyus, Nishihara, Okinawa 903‑0213 Japan

Correction to: *Scientific Reports* 10.1038/s41598-022-16024-6, published online 27 July 2022

The original version of this Article contained an error. In the Results section, under the subheading ‘Survival and growth rate of juvenile (field experiment)’,

“In both 2015 and 2016, most juveniles in exposed orientation at 3–5 and 20 m did not survive the first month, with no juveniles surviving past four months (Fig. 3).”

now reads:

“Most juveniles in exposed orientation at 3–5 m (in 2015, 2016) and 20 m (in 2016) did not survive the first month (Fig. 3).”

The original Article has been corrected.

