# Supplementary material for: Limited acclimation of early life stages of the coral Seriatopora hystrix from mesophotic depth to shallow reefs
Source: Sci Rep. 2022 Jul 27;12:12836. doi: 10.1038/s41598-022-16024-6 (PMC9329301; doi:10.1038/s41598-022-16024-6)
Supplement: Supplementary file 1 — Supplementary Information. [file 41598_2022_16024_MOESM1_ESM.pdf]

Supplementary Information

Limited acclimation of early life stages of the coral *Seriatopora hystrix* from mesophotic depth to shallow reefs

Rian Prasetia[1], Frederic Sinniger[1], Takashi Nakamura[1,2], Saki Harii[1,*]

[1]Sesoko Station, Tropical Biosphere Research Center, University of the Ryukyus, Sesoko 3422, Motobu, Okinawa 905-0227, Japan

Science Drive 4, 117558, Singapore

[2]Faculty of Science, University of the Ryukyus, Nishihara, Okinawa 903-0213, Japan

*Corresponding author: Saki Harii, sharii@lab.u-ryukyu.ac.jp

Supplementary Materials and Methods

1. Environmental measurements

At each depth and frame orientation (i.e., exposed and shaded orientation) in the study sites, light intensity and seawater temperature were measured hourly using light and temperature loggers (HOBO Pendant/Light Data Logger, Onset Computer Corporation, USA). The loggers were replaced for data collection monthly. Particularly for light loggers, only light data from the first 2 weeks of instalment were used for the analysis since algae and sediment often cover the logger sensor. Light intensity data were converted from Lux to PAR (Photosynthetically Active Radiation, between 400 and 700 nm) units (µmol quanta·m^{-2}·s^{-1}) using a conversion factor of 0.0184 [1]. To confirm the light intensity from HOBO light loggers,

PAR light meters (Compact LW and DEFI-2L, JFE Advantech Co. Ltd., Japan) were also deployed several times during the experiments.

2. Maximum quantum yield of juveniles

Rapid light curves (RLCs) using the saturating pulse method were conducted on each coral juvenile to measure maximum quantum yield *(Fv/Fm*) and maximum relative electron transport rate (rETRmax). Before dark adapted, the algae that grew surrounding the juveniles were cleaned to avoid algal effect on the measurement. The minimum fluorescence (*Fo*) of the chlorophyll was determined using 3 μsecond pulses (peak emission at 650 nm) and the maximum fluorescence (*Fm*) was measured after a 0.8 second saturating light pulse (~10,000 μmol quanta$\cdot m^{-2} \cdot s^{-1}$). The dark-adapted maximum quantum yield [$Fv/Fm = (Fm-Fo)/Fm$)] was then measured. Subsequent saturation light pulses (41, 102, 237, 431, 631, 863, 1297, and 1774 μmol quanta$\cdot m^{-2} \cdot s^{-1}$) every 15 second per step, with gain = 6, were applied to obtain *Fv`/Fm`* (effective quantum yield of Photosystem II) to calculate maximum relative electron transport rate [rETRmax = *Fv`/Fm* · PAR · 0.5].

3. Juvenile tissue extracts for algal density and chlorophyll pigments analyses

Soft tissues of each juvenile were centrifuged to isolate the symbionts from the host tissue. The pellets were used for algal density and chlorophyll pigments analyses (described in [2]), while the supernatants were stored at -80 ºC for fluorescent protein analyses (see "*Characteristics and spectral properties of fluorescent protein of juveniles"*). Data were normalized to algal density and are reported in chlorophyll pigments (*a* and c_2) per cell and per surface area and the chlorophyll (a:c_2) ratio. The juvenile surface area (SA) was estimated using the equation for a conic shape:

$$SA = \mu \cdot (d/2) \cdot h$$

where geometric diameter (d) and height (h) of the juveniles were measured under a dissecting microscope.

4. Characteristics and spectral properties of fluorescent proteins of juveniles

The presence and absence of host fluorescence was observed on living juveniles (in 2016 experiment) under a dissecting microscope by using a blue light source with a yellow-barrier filter (excitation 440-460 nm, emission ~500 nm, NightSea, USA). In order to examine spectral properties of fluorescent proteins, frozen juvenile tissue samples were gradually thawed in a container with ice. The tissue samples (200 μl; 2 replicates) were then measured using a microplate reader (SH-9000; Corona Electric Co., Hitachinaka, Japan) based on [3]. Fluorescent proteins were classified according to [4].

Supplementary Results

Fluorescent protein of juveniles

The juveniles at 40 and 20 m depths in the 2016 experiment (regardless of the orientation) displayed green fluorescence attributed to a green fluorescent protein (GFP). GFP excitation peaked at about 478 nm, and the emission peaked at around 499-502 nm. The GFP was observed in the tentacles and oral discs of the polyps.

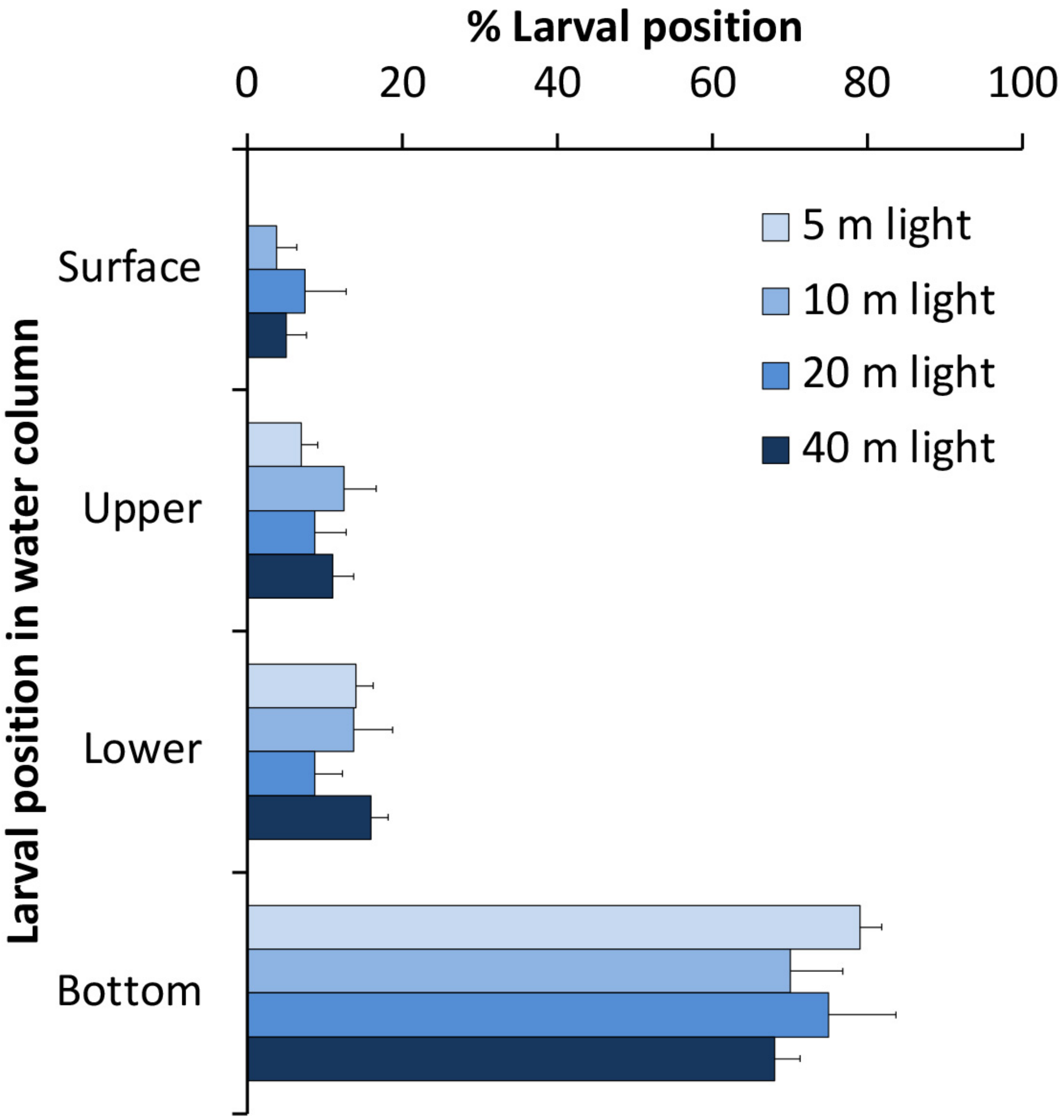

Supplementary Figure 1 Percentage of mesophotic *S. hystrix* larvae at different positions in the water column under four depth light conditions (mean ± SE; n = 10 for 5 and 40 m light; n = 8 for 10 and 20 m light).

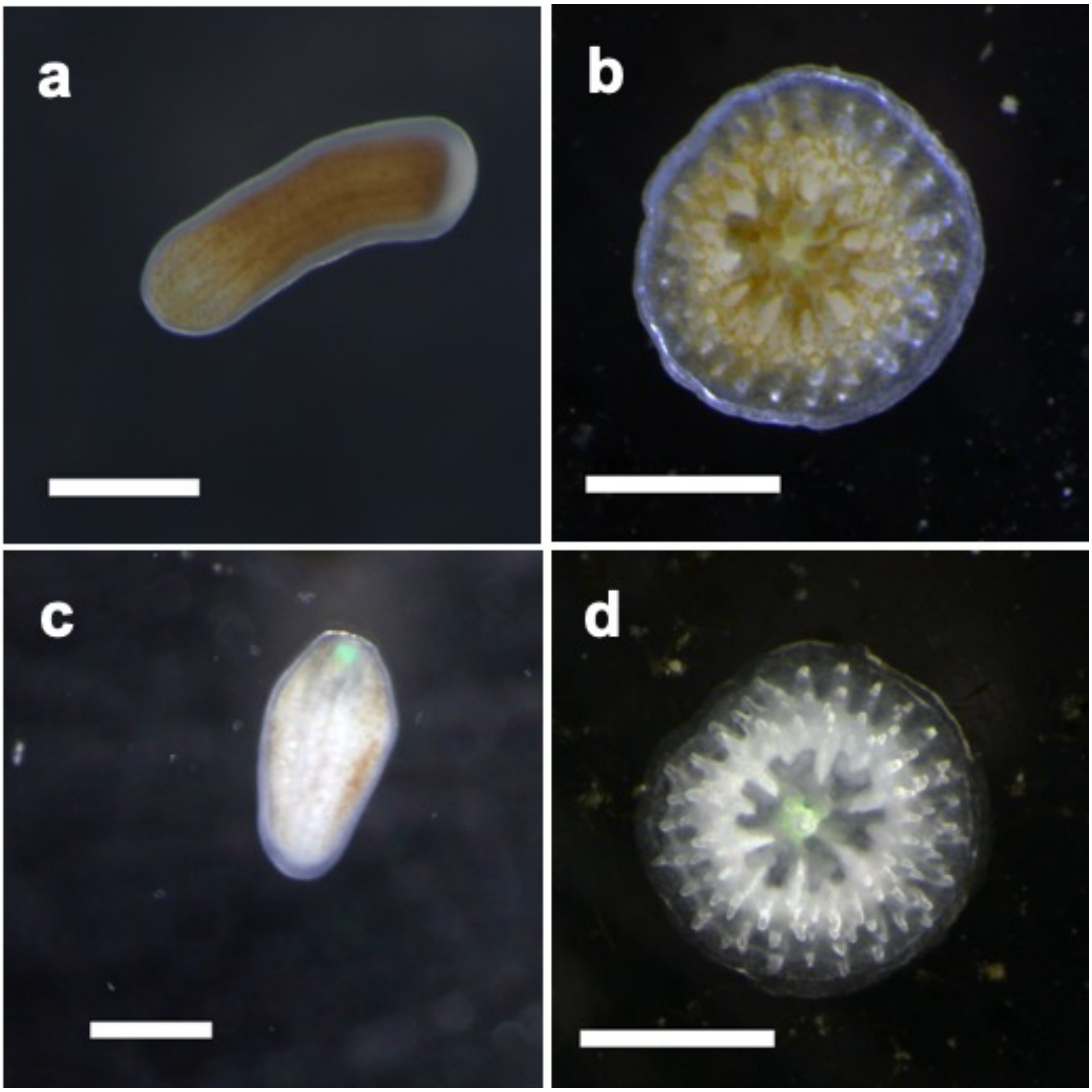

Supplementary Figure 2 Mesophotic *S. hystrix* larvae defined as healthy swimming (a) and settled (b) larvae at control light (40 m) condition. Under 5 m light condition, the swimming (c) and settled (d) larvae were pale/bleached. Scale bars = 400 μm.

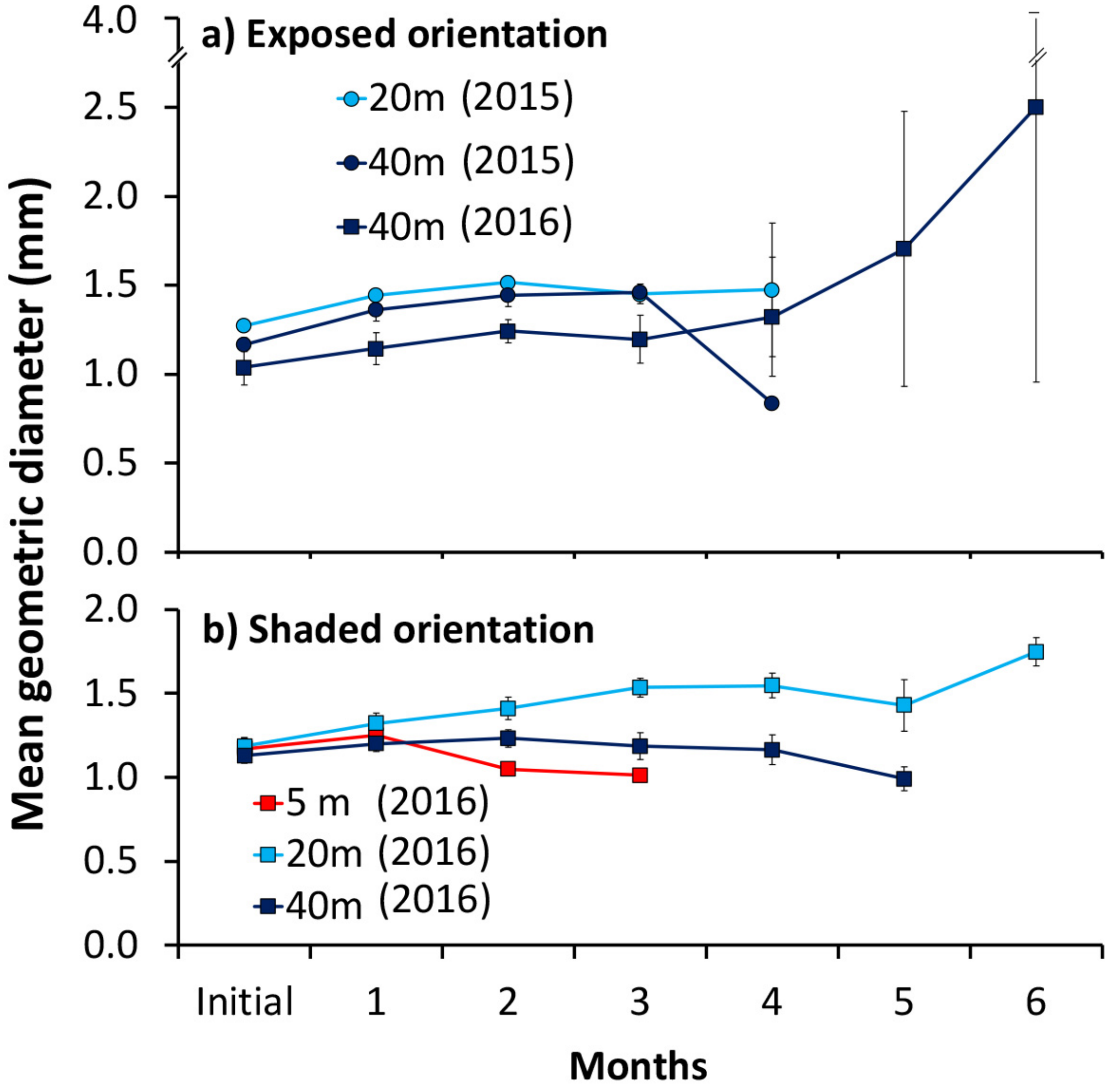

Supplementary Figure 3 Mean geometric diameter of *S. hystrix* per month at exposed (a) and shaded (b) orientation in 2015 experiment from August 2015 to February 2016 (marked with circles) and in 2016 experiment from August 2016 to February 2017 (marked with square boxes) (mean ± SE).

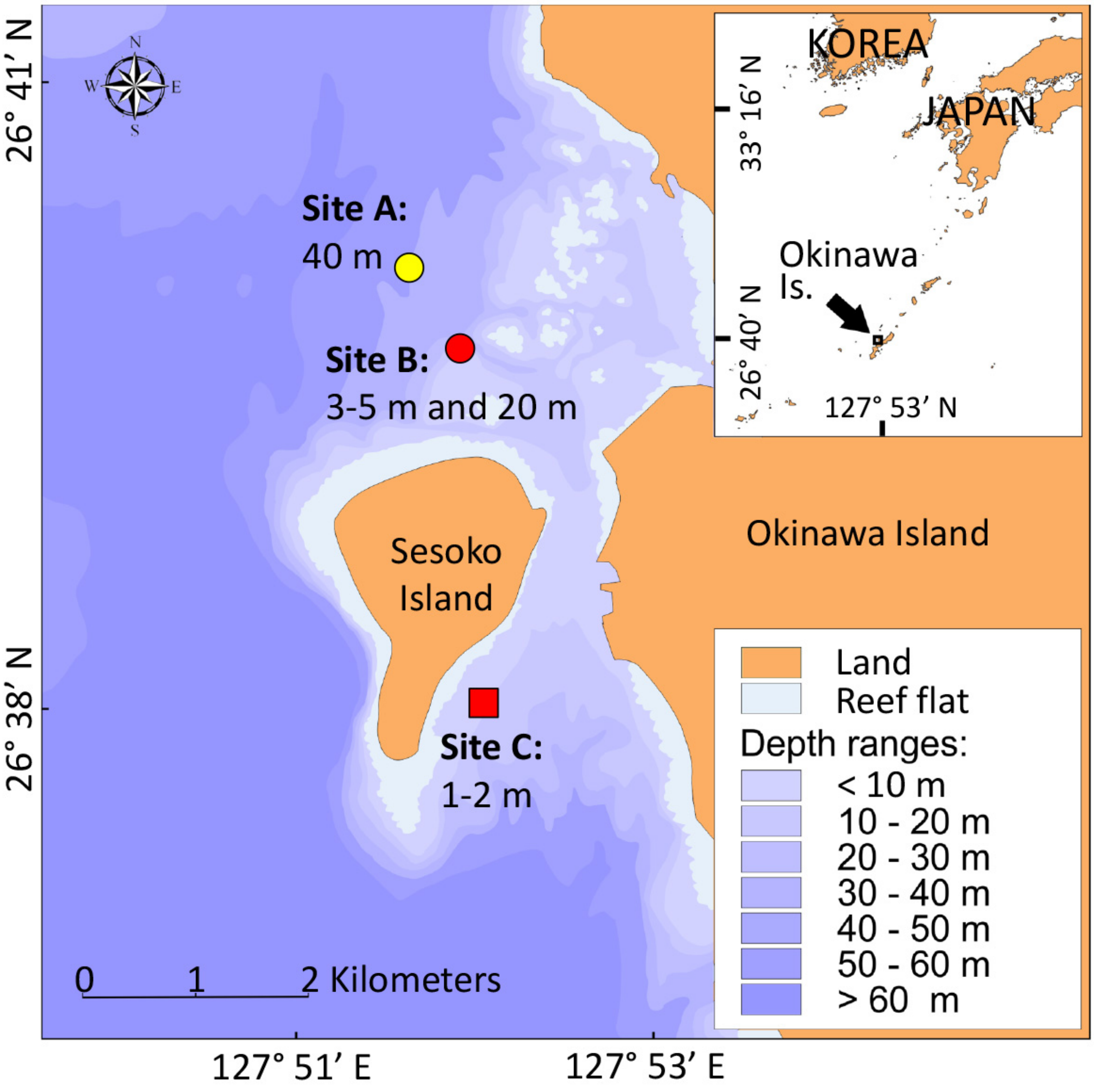

Supplementary Figure 4 Map of the studied sites. Newly settled juveniles, light intensity and seawater temperature loggers were deployed at the Site A at 40 m (a yellow circle) and the Site B at 3-5 m and 20 m (a red circle) northern Sesoko Island in 2015 and 2016. An additional temperature logger was deployed at the Site C at 1-2 m southern Sesoko Island (a red square) in 2016. Map from Japan Coast Guards [5] redrawn using CorelDraw V. 16.0 (www.coreldraw.com/en/pages/coreldraw-x6/).

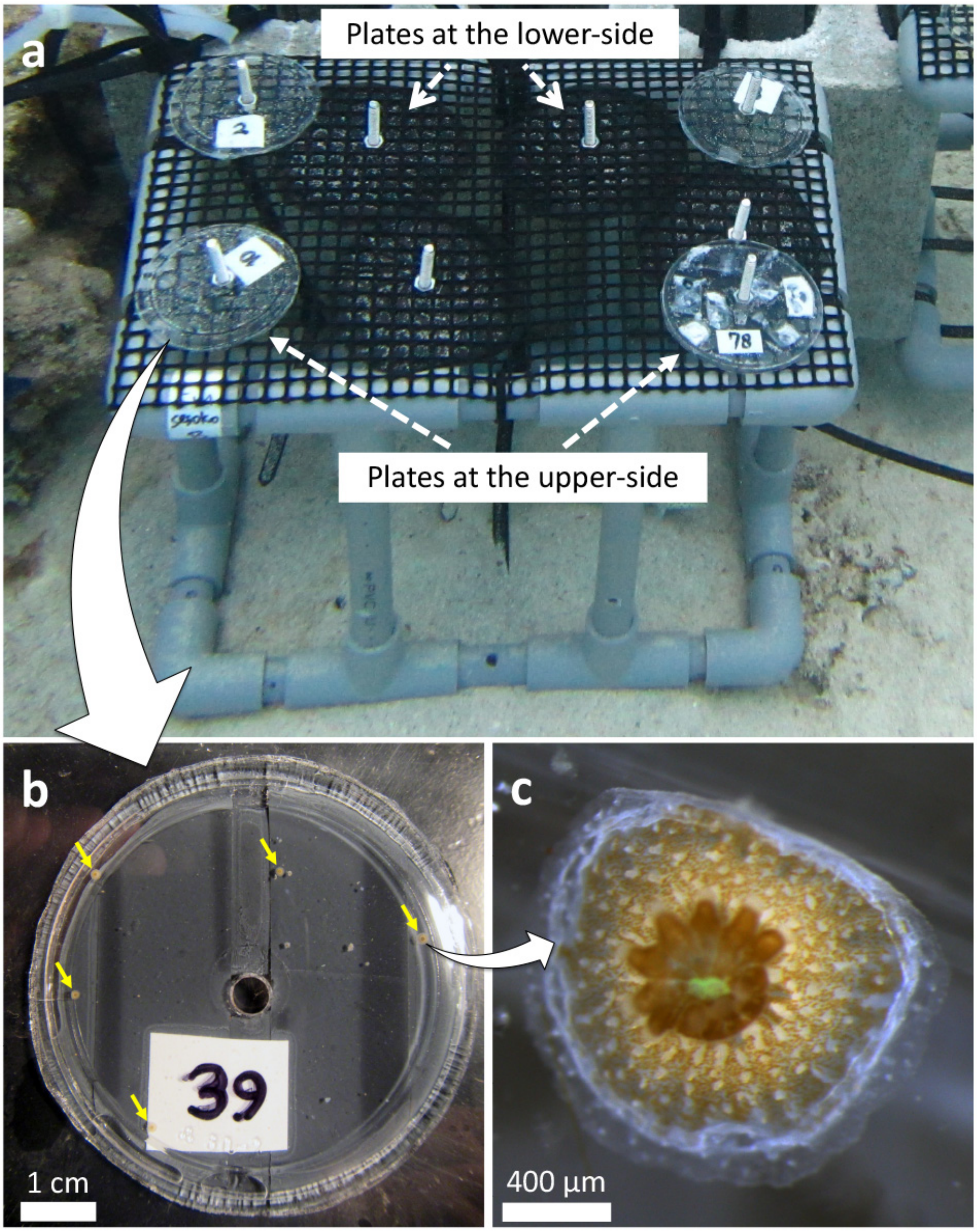

Supplementary Figure 5 A moveable rectangular frame used where the plastic settlement plates were placed at the exposed and shaded orientation (a). The juveniles of *S. hystrix* (yellow arrows) settled on the plastic plates (b-c).

Supplementary Table 1 Pair-wise comparisons of K-M survival estimates using Mantel-cox log rank tests for survival of juveniles in 2015 and 2016 comparing different depths (3-5, 20 and 40 m) and orientations (exposed and shaded). Significant differences are shown in bold. NA= test result was not applicable.

Depth/orientation	3-5 m Exposed	3-5 m Shaded	20 m Exposed	20 m Shaded	40 m Exposed
2015					
20 m Exposed	**<0.001**				
40 m Exposed	**<0.001**		**0.005**		
2016					
3-5 m Shaded	**<0.001**				
20 m Exposed	NA	**<0.001**			
20 m Shaded	**<0.001**	**0.003**	**<0.001**		
40 m Exposed	**<0.001**	0.505	**0.001**	0.071	
40 m Shaded	**<0.001**	**0.005**	**<0.001**	0.138	0.444

Supplementary Table 2 Summary of Welch's t-test for *Fv/Fm,* rETRmax, algal density, Chlorophyll *a* and c_2 per surface area, Chlorophyll *a* and c_2 per cell and Mann-Whitney U-test for Chl a:c_2 ratio (marked by *) between 20 m and 40 m depths in 2015. n.a.= post hoc test was not applicable.

	df1	df2	*P*	Pairwise comparisons
Fv/Fm	1	4.538	**0.049**	20 m < 40 m
rETRmax	1	5.498	0.898	n.a
Algal density	1	6.963	0.795	n.a
Chl *a* per surface area	1	6.981	0.535	n.a
Chl c_2 per surface area	1	6.485	0.598	n.a
Chl *a* per cell	1	7.672	0.074	n.a
Chl c_2 per cell	1	5.254	0.785	n.a
Chl a:c_2 ratio*	-	-	0.548	n.a

Supplementary Table 3 Number of colonies sampled and used for larval behavior and settlement (laboratory experiment) and juvenile acclimation (field experiment) in 2015 and 2016.

Year	Date	No of colonies sampled	No of colonies used for experiments	
			Larval behavior and settlement (laboratory exp.)	Juvenile acclimation (field exp.)
2015	Jun 23	10	3	-
	Jun 29	6	1	-
	July 24	10	-	8
2016	July 11	9	5	9

Supplementary Table 4 Light intensity and spectral quality used for larval behaviour and settlement experiments representing the native light environment at 5 m, 10 m, 20 m, and 40 m depths.

Light environment (depth)	Light intensity (μmol quanta m^{-2} s^{-1})	Light spectral quality
5 m	50-60	Full light spectrum
10 m	250	Full light spectrum
20 m	450	Full light spectrum without UV and red light
40 m	600	Blue light spectrum

References

1. Thimijan, R. W. & Heins, R. D. Photometric, radiometric, and quantum light units of measure: A review of procedures for interconversion. *Hortic. Sci.* **18**, 818–820 (1983).
2. Nakamura, T., van Woesik, R. & Yamasaki, H. Photoinhibition of photosynthesis is reduced by water flow in the reef-building coral *Acropora digitifera*. *Mar. Ecol. Prog. Ser.* **301**, 109–118 (2005).
3. Roth, M. S. *et al.* Fluorescent proteins in dominant mesophotic reef-building corals. *Mar. Ecol. Prog. Ser.* **521**, 63–79 (2015).
4. Alieva, N. O. *et al.* Diversity and evolution of coral fluorescent proteins. *PLoS One* **3**, e2680 (2008).
5. Japan Coast Guard. Bathymetric chart: Ie Sima, Okinawa Sima, Nansei Syoto, Nippon [Map]. 1: 50,000. *No 6507*. (1987).
